# Tuberculosis reactivation during novel, biologic therapy

**DOI:** 10.1186/1471-2334-13-S1-O25

**Published:** 2013-12-16

**Authors:** Cristina Popescu, Violeta Molagic, Cătălin Tilişcan, Ruxandra Moroti, Adriana Hristea, Mihaela Rădulescu, Raluca Mihăilescu, Daniela Munteanu, Iulia Niculescu, Raluca Năstase, Raluca Dulama, Alina Lobodan, Raluca Hrişcă, Raluca Jipa, Anca-Ruxandra Negru, Irina Lăpădat, Roxana Petre, Mircea Chiotan, Cornel Camburu, Viorica Poghirc, Raluca Popescu, Victoria Aramă

**Affiliations:** 1 National Institute for Infectious Diseases “Prof. Dr. Matei Balş”, Bucharest, Romania; 2 Carol Davila University of Medicine and Pharmacy, Bucharest, Romania

## Background

Biologic immunotherapy such as anti-TNF or monoclonal antibody against CD20 protein revolutionized the treatment of autoimmune diseases. Unfortunately, the patients receiving this therapy are at higher risk for bacterial or fungal infections, and at risk for reactivation of latent viral infections and latent tuberculosis. We present a series of patients receiving biologic therapy, admitted in the Adults 3 Department of the National Institute for Infectious Diseases “Prof. Dr. Matei Balş” for prolonged fever.

## Case report

All of the patients were diagnosed with tuberculosis. One patient was treated with rituximab for non-Hodgkin lymphoma, 2 patients were treated with infliximab for rheumatoid arthritis, one patient was treated with infliximab for ankylosing spondylitis and one patient was treated with etanercept for severe psoriasis. Two of the patients were diagnosed with pulmonary tuberculosis and for three patients the localization of tuberculosis was not identified. All of the patients had quantiferon TB gold positive. Only one of the patients had positive microbiologic tests for tuberculosis. All of the patients had a good therapeutic response after anti-tuberculous therapy. None of the patients had been tested for latent tuberculosis prior to initiation of biologic therapy.

## Conclusion

We emphasize the importance of anti-tuberculosis prophylaxis for patients diagnosed with latent tuberculosis, before the start of biologic therapy. The diagnosis of latent tuberculosis remains an important issue especially in a country with high incidence of tuberculosis.

